# Changes in the structure of the microbial community within the phycospheric microenvironment and potential biogeochemical effects induced in the demise stage of green tides caused by *Ulva prolifera*

**DOI:** 10.3389/fmicb.2024.1507660

**Published:** 2024-11-05

**Authors:** Xiaoxue Liu, Yu Zang, Shiliang Fan, Xiaoxiang Miao, Mingzhu Fu, Xiaojun Ma, Mei Li, Xuelei Zhang, Zongling Wang, Jie Xiao

**Affiliations:** ^1^Research Center of Marine Ecology, First Institute of Oceanography, MNR, Qingdao, China; ^2^Laboratory for Marine Ecology and Environment Science, Laoshan Laboratory, Qingdao, China; ^3^College of Environmental Science and Engineering, Ocean University of China, Qingdao, China

**Keywords:** harmful algal blooms, phycosphere, microorganisms, element cycle, metagenomic

## Abstract

Green tides caused by *Ulva prolifera* occur annually in the Yellow Sea of China, and the massive amount of biomass decomposing during the demise stage of this green tide has deleterious ecological effects. Although microorganisms are considered key factors influencing algal bloom demise, an understanding of the microbial-algae interactions within the phycospheric microenvironment during this process is still lacking. Here, we focused on the variations in phycospheric microbial communities during the late stage of the green tide in three typically affected areas of the Yellow Sea via metagenomic sequencing analysis. In total, 16.9 million reads obtained from 18 metagenome samples were incorporated into the assembled contigs (13.4 Gbp). The phycosphere microbial community composition and diversity changed visibly during the demise of *U. prolifera*. The abundances of algae-lysing bacteria, Flavobacteriaceae at the family level and *Alteromonas*, *Maribacter*, and *Vibrio* at the genus level increased significantly in the phycosphere. In addition, the levels of glycoside hydrolases (GHs) and polysaccharide lyases (PLs) enzymes, which decompose *U. prolifera* polysaccharides in the phycosphere, were greater. Therefore, the degradation of algal polysaccharides can increase the efficiency of carbon metabolism pathways in the phycospheric microenvironment. Most of the genes detected in the phycosphere, especially *norC, nrfA*, and *nasA*, were associated with nitrogen metabolism pathways and showed dynamics related to the demise of the large amount of organic matter released by a green tide. Therefore, the demise of green tide algae may affect the potential carbon and nitrogen cycles of the phycospheric microenvironment by driving changes in the structure and diversity of microbial communities. Our research provides a novel perspective to better understand the ecological impact of *U. prolifera* during the green tide demise stage.

## 1 Introduction

Green tides refer to harmful algal bloom (HAB) phenomena caused by the explosive proliferation or high accumulation of some green algae species under specific environmental conditions, resulting in substantial global marine environmental problems (Rybak, 2018). Since 2007, the green tide in the Yellow Sea of China caused by *Ulva prolifera* has occurred annually (Xiao et al., 2020; Yuan et al., 2022) and is considered the largest green tide outbreak event in the world (Zhao et al., 2019). The green tide typically forms in the South Yellow Sea around mid-April every year and subsequently drifts northward, propelled by ocean currents and monsoons, finally reaching its peak distribution and coverage area in the sea of Shandong Province by June (Xiao et al., 2013). As the seawater temperature increases and the nutrient salt content decreases by late July, the buoyancy, activity, and photosynthetic rates of the alga plummet, indicating that the floating green algae are demising. Only a fraction of *U. prolifera* are collected before they settle to the bottom of the ocean and demise in Shandong Province, off the shore of the cities of Qingdao, Rizhao, Weihai, etc. The demise of *U. prolifera* results in the release of large amounts of nitrogen and phosphorus nutrients that are absorbed during its migration across the Yellow Sea (Wang Z. et al., 2023). This process not only has a substantial effect on the socioeconomic development of coastal regions but also has irreversible ecological repercussions on marine ecosystems (Bastos et al., 2019; Li et al., 2019; Wang et al., 2021).

In algal ecosystems, the concept of the “phycosphere” is similar to that of the plant “rhizosphere” and refers to the microenvironment immediately surrounding an algae cell that is enriched in organic molecules exuded by the cell into the surrounding water (Seymour et al., 2017). Studies have demonstrated that phycospheric microorganisms interact with algae in multiple ways during the different stages of algae growth, forming symbiotic, competitive, and harmful relationships with them (Daly et al., 2022; Zhao et al., 2023). As hosts, algae provide habitats, vitamins, inorganic salts and carbohydrates for phycospheric microorganisms (Krug et al., 2020). In contrast, phycospheric microorganisms provide amino acids, polypeptides, nucleotides, fatty acids and dimethylsulfoniopropionate for algae to promote their growth (Caruana and Malin, 2014; Variem and Kizhakkedath, 2021). Therefore, the phycospheric microenvironment provides a key medium for the interaction between algae and microorganisms (Noreen et al., 2023), and can facilitate the colonization of specific microorganisms within it (Han et al., 2021). This microenvironment also includes many algae-lysing bacteria, which can cleave algal bloom-causing species through physical contact or secrete algae-lysing substances (including proteases, alkaloids, benzoic acid, amino acids, etc.) and compete for nitrogen and phosphorus, promoting the reduction of HABs (Nair et al., 2022; Thoré et al., 2023). For example, *Saprospira* can directly destroy the diatom cell wall, leading to algal cell dissolution and death (Wang M. et al., 2020).

Although interactions between algae and bacteria occur in the microscale environment, they influence the entire process of HABs and can also drive and affect the cycle of marine elements in the outbreak area. The large amount of dissolved organic carbon (DOC) secreted by algae provides support for the rapid growth and reproduction of phycospheric microorganisms during the early stage of HABs (Piontek et al., 2011). The DOC released by algae is composed mainly of polysaccharides, and phycospheric microorganisms also use polysaccharides in a variety of ways. For example, *Flavobacterium* mainly ingests and degrades complex polysaccharides and other high-molecular-weight organic carbon, while Alteromonadaceae can extract organic carbon by degrading polysaccharides or monosaccharide types with low complexity (Qu et al., 2021). Furthermore, dissolved organic nitrogen (DON) and dissolved organic phosphorus (DOP) are remineralized by phycospheric microorganisms, providing substantial nutrients for algae (Krug et al., 2020; Zhao et al., 2023). When the HABs enter the late stage, the number of algae begin to decrease, and the change in algae source material not only affects the composition of the phycospheric microbial community but also changes the content of biogenic elements in the ocean. For example, heterotrophic bacteria in the phycosphere have a great effect on the degradation and remineralization of organic matter during the late stage of diatom blooms, thus significantly affect the marine carbon cycle (Buchan et al., 2014; Wu et al., 2023). Thus, the relationship between algae and microorganisms plays a crucial role in regulating biogeochemical element cycles in the phycospheric microenvironment.

The microbial community can exhibit obvious specificity during HABs, and the different development stages of HABs affect the structural variation and element cycles of the microbial community (Ma et al., 2024). For example, Bacteroidetes and Proteobacteria dominate the microbial communities in red tide areas, whereas Proteobacteria dominate in non-red tide areas (Delmont et al., 2014). After a dinoflagellate bloom (*Gymnodinium catenatum*) enters the outbreak stage, the microbial abundance involved in the reduction of the citrate cycle, carbon degradation and nitrification transport increases significantly (Zhou et al., 2020). During the extinction period of algal blooms, the majority of bacteria are *Pseudomonas* and *Bacillus*, which may have denitrification functions (Zheng et al., 2008). Because of the complex relationship between microorganisms and algae, it is widely believed that the beginning, persistence and disappearance of HABs involve interactions with microbial communities (Qu et al., 2021). Therefore, we focused on a primary site associated with the demise of green tides caused by *U. prolifera* in the Yellow Sea, and the composition structure, functional changes and related gene functional groups of microbial communities in seawater were analyzed via metagenomic technology coupled with environmental physicochemical factors. Our primary objectives were to explore (1) the changes in microbial community structure composition during the demise stage of *U. prolifera* green tides and (2) the element cycles that microorganisms participated in the phycospheric microenvironment. This research is crucial for a comprehensive understanding of the ecological effects exerted by phycospheric microorganisms during the green tide demise period.

## 2 Materials and methods

### 2.1 Samples collection

In early June 2023, *U. prolifera* green tide began to cross 35 degrees north latitude and entered the coastal of Shandong Province. The green tide covered a maximum area of 998 square kilometers on June 25. Since then, the green tide entered the demise stage. Therefore, according to satellite remote sensing data of *U. prolifera* green tide, we chose three cities most seriously affected by the late-stage green tide on the coast of the Shandong Province, Qingdao (36.05°E, 120.43°N), Rizhao (35.56°E, 119.66°N), and Haiyang (36.71°E, 121.31°N) to set sampling stations from July 15 to 25, 2023.

The methods of seawater samples collection according to previous study (Qu et al., 2023). The phycospheric seawater samples (QDP1-3, RZP1-3, and HYP1-3) were collected from the center of the green algal mats (the average distance from the thalli was less than 5 mm) in the three sampling stations. And the control area samples were collected from nonalgae-covered seawater (QDN1-3, RZN1-3, and HYN1-3) in the three sampling stations. All of the seawater samples were collected from a 0–20 cm depth using a sterilized hydrophore. A total of 1.5 L of seawater was prefiltered through a 3-μm pore size polycarbonate filter, and then the seawater microorganisms were filtered through a 0.22-mm pore size polycarbonate filter. All of the filters were stored at −80°C until DNA extraction.

### 2.2 Measurement of environmental factors

The basic physical and chemical characteristics of the phycospheric seawater and nonalgae-covered seawater, including temperature, dissolved oxygen (DO), pH, and salinity, were measured using a YSI ProQuatro handheld multiparameter meter (Xylem, Inc., Yellow Springs, OH, United States). The concentration of DOC was analyzed via a Multi N/C 3100 Analyser (Analytik Jena AG, Jena, Germany). The concentrations of NO_3_^−^ and NO_2_^−^ were determined via the cadmium-copper reduction method and the standard pink azo dye method, respectively. The concentration of NH_4_^+^ was determined via the indophenol blue method, and PO_4_^3−^ was determined via the molybdate blue method. Dissolved inorganic nitrogen (DIN) was calculated as the sum of the concentrations of NO_3_^−^, NO_2_^−^, and NH_4_^+^, and dissolved inorganic phosphorus (DIP) was calculated as the concentration of PO_4_^3−^. The total dissolved nitrogen (TDN) and phosphorus (TDP) contents were determined through persulfate oxidation via AAIII. DON and DOP concentrations were calculated as the differences between TDN and DIN and between TDP and DIP, respectively (Zhang et al., 2020).

### 2.3 DNA extraction and metagenomic sequencing

Total DNA was extracted from the filtered samples using a FastDNA spin kit following the manufacturer’s instructions. The concentration and purity of the extracted DNA were assessed using a TBS-380 Mini-Fluorometer (Turner Biosystems, Sunnyvale, United States) and a NanoDrop 2000 Spectrophotometer (Thermo Fisher Scientific, MA, United States), respectively. The quality of the extracted DNA was evaluated via a 1% agarose gel, ensuring that the OD260/OD230 ratio was greater than 1.8. Covaris M220 (Gene Company Limited, China) was used to fragment the extracted DNA to an average size of ~400 bp, after which a paired-end library was constructed via NEXTflex^™^ Rapid DNA-Seq (Bioo Scientific, Austin, TX, United States). Adapters containing the full complement of sequencing primer hybridization sites were ligated to the blunt ends of the fragments. Paired-end sequencing was performed via Illumina NovaSeq Reagent Kits at Majorbio Bio-Pharm Technology Co. (Shanghai, China).

### 2.4 Sequence quality control and assembly

The raw reads from metagenomic sequencing were processed to generate clean reads, adaptor sequences were removed, and low-quality reads (defined as reads containing N bases, with a minimum length threshold of 50 bp and a minimum quality threshold of 20) were trimmed using fastp (version 0.20.0)^1^ (Chen et al., 2018). The host chloroplast genome was subsequently eliminated via BWA (version 0.7.17). The resulting high-quality reads were then assembled into contigs with MEGAHIT (version 1.1.2) (Li et al., 2015), with contigs measuring 300 bp or longer selected as the final assembly results.

### 2.5 Gene prediction and annotation

Open reading frames (ORFs) within contigs were identified using MetaGene. A nonredundant gene catalog was constructed via CD-HIT (version 4.6.1) with a sequence identity and coverage threshold of 90% (Fu et al., 2012). After quality control, the reads were mapped to the nonredundant gene catalog with 95% identity using SOAPaligner, and the gene abundance in each sample was determined. Gene annotation was performed using BLASTP (Buchfink et al., 2015) and the Kyoto Encyclopedia of Genes and Genomes (KEGG) database (release 84.1) (Kanehisa and Goto, 2000) for functional and taxonomic analysis, and only the best hits were retained.

### 2.6 Bioinformatics and statistical analysis

Alpha diversity was assessed by computing the Shannon and Chao indices via Mothur (version 1.30.2). The bacterial community composition and functional differences were visualized using principal coordinate analysis (PCoA) on the basis of the Bray-Curtis distance (Dixon, 2003). The top 15 taxa at the genus level were compared using the Wilcoxon rank-sum test. Network modular analysis was conducted using Gephi 0.10.1 (Bastian et al., 2009), and Spearman’s rank correlation coefficient with a threshold of 0.5 was used. Biogeochemical element cycle enrichment analysis was performed via the DESeq2 package in R (log2count function) for the gene annotation data, and the abundance of genes across different samples during the demise of green tide was compared. One-way ANOVA was performed using the multcomp tool of the R package. Combined with the KEGG database and published literature information (Zhou et al., 2020), marker gene sets related to the carbon cycle, nitrogen cycle, sulfur cycle and phosphorus cycle were constructed. The Mantel test (Legendre and Fortin, 2010) was used to assess the correlation between the community distance matrix and the environmental variable distance matrix, which was conducted via QIIME on the basis of Bray-Curtis distance. Pearson’s coefficient was used to calculate the correlation between functional enzymes or genes and environmental factors, and a numerical matrix heatmap was drawn in R (pheatmap package).

## 3 Results

### 3.1 Variations in environmental factors in the phycosphere

**Figure 1:**
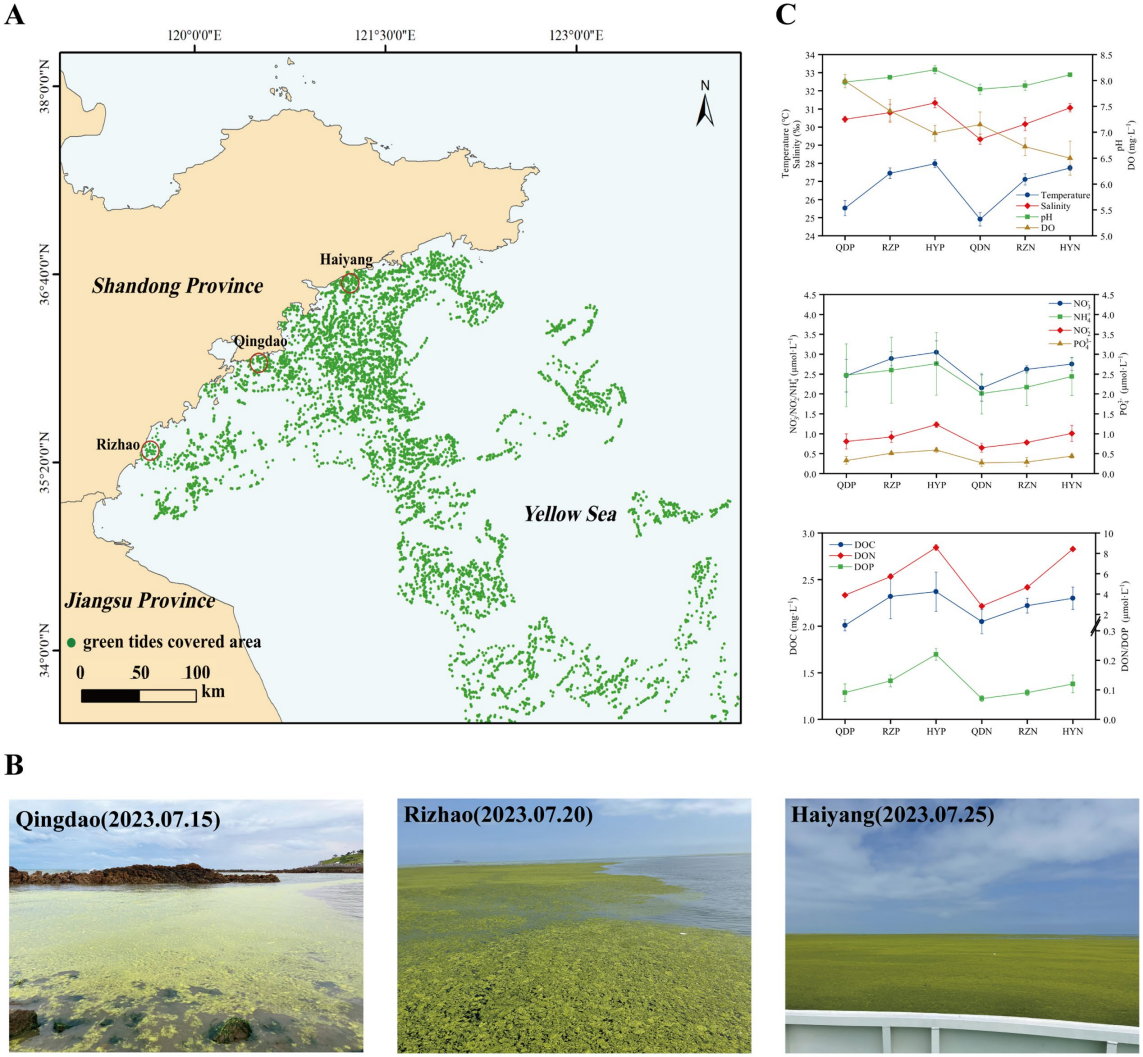
Sampling sites: setting information and environmental factors. **(A)** Locations of the sampling sites (red circles) in the coastal cities of the Yellow Sea, China. **(B)** Field conditions of the sampling sites. **(C)** The environmental factors of the sampling sites. The distribution data of the green tides were obtained from the interpretation and analysis of remote sensing satellite HY-1D (CZI) data. The imaging time was 2023-07-20, with a resolution of 50 m.

We analyzed the variation in environmental factors at different stations during the late stage of the green tide separately for phycospheric seawater and nonalgae-covered seawater (Figure 1). The concentration of DO in phycospheric seawater was significantly greater than that in the nonalgae-covered seawater. The average concentration of DOC in the phycospheric seawater was 2.23 mg·L^−1^, which was higher than that in the nonalgae-covered seawater (2.17 mg·L^−1^). Additionally, the primary distinguishing environmental factor between the two seawater areas was the concentration of nutrients. The concentrations of NO_3_^−^, NO_2_^−^, and NH_4_^+^ all tended to increase in the two seawater areas, but the concentrations in the phycospheric seawater were significantly greater. Moreover, the DON concentrations in the phycospheric seawater increased by 14.53% compared to the nonalgae-covered seawater. In phycospheric seawater DIP and DOP concentrations varied from 0.23–0.65 μmol/L and 0.06–0.24 μmol/L, respectively, significantly higher than that in the nonalgae-covered seawater.

### 3.2 Variations in the microbial community structure and diversity in the phycosphere

In our research, 16.9 million reads were incorporated into the assembled contigs (13.4 Gbp) after quality control and host removal (Supplementary Table 1). According to the metagenomic experimental taxonomic species statistics, a total of 90.28% bacteria, 5.38% viruses, 2.52% archaea and 1.81% eukaryotes were recorded in our experiment. On the basis of the nonredundant (NR) database, we annotated bacteria across 54 phyla, 104 classes, 193 orders, 385 families and 980 genera.

**Figure 2:**
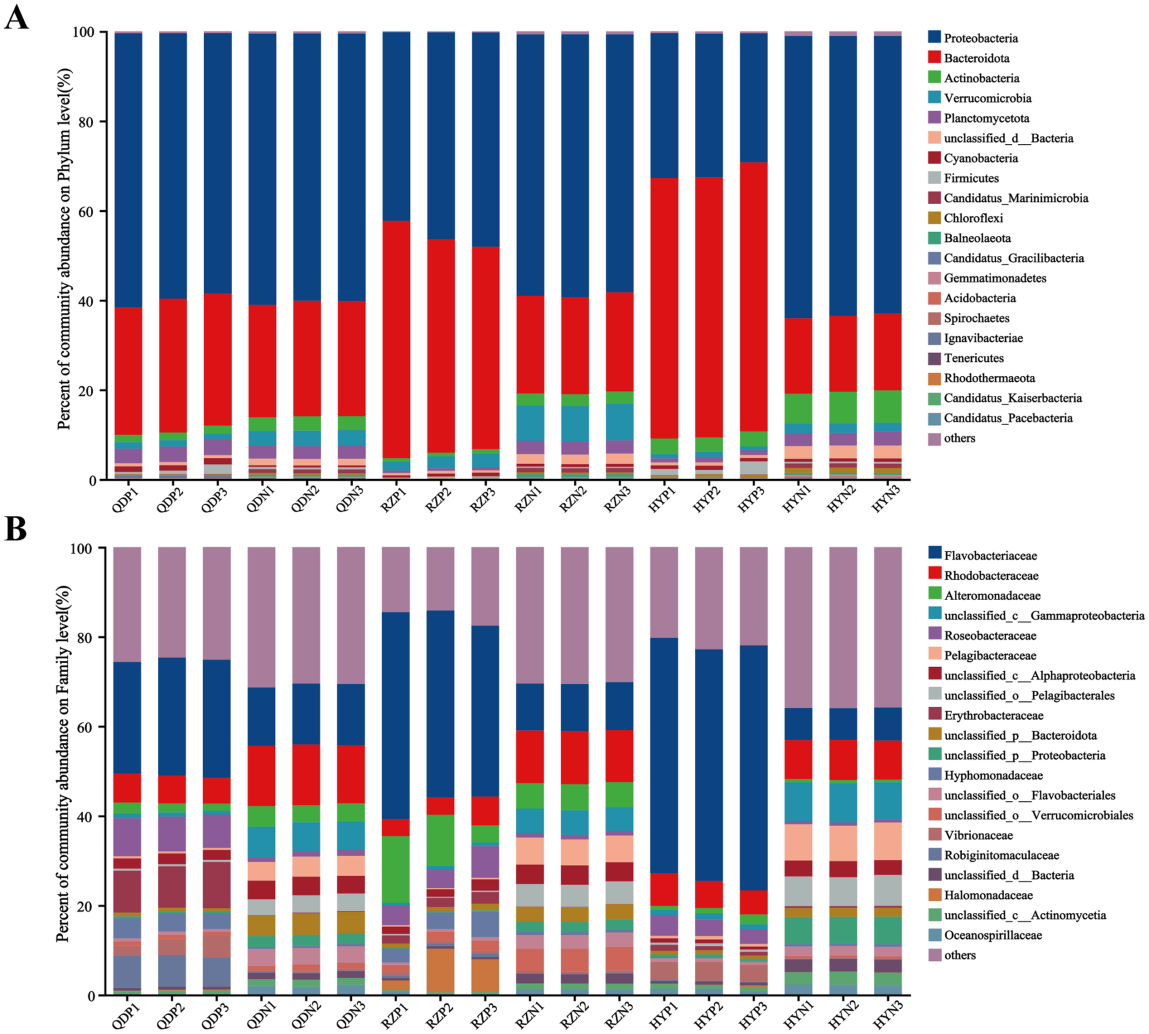
Relative abundance of bacteria at the phylum **(A)** and family **(B)** levels.

At the phylum level, Proteobacteria (with a relative abundance ranging from 32.05 to 63.28%) and Bacteroidota (17.36–58.08%) dominated the bacterial community across the samples (Figure 2). The abundances of Bacteroidota, Cyanobacteria and Candidatus_Gracilibacteria were greater in the phycospheric seawater microorganism groups (QDP, RZP and HYP) during the demise phase. At the family level, Flavobacteriaceae (26.40–51.70%), Rhodobacteraceae (3.97–6.18%) and Alteromonadaceae (1.17–11.32%) were present in greater proportions in the phycospheric seawater microorganism group.

**Figure 3:**
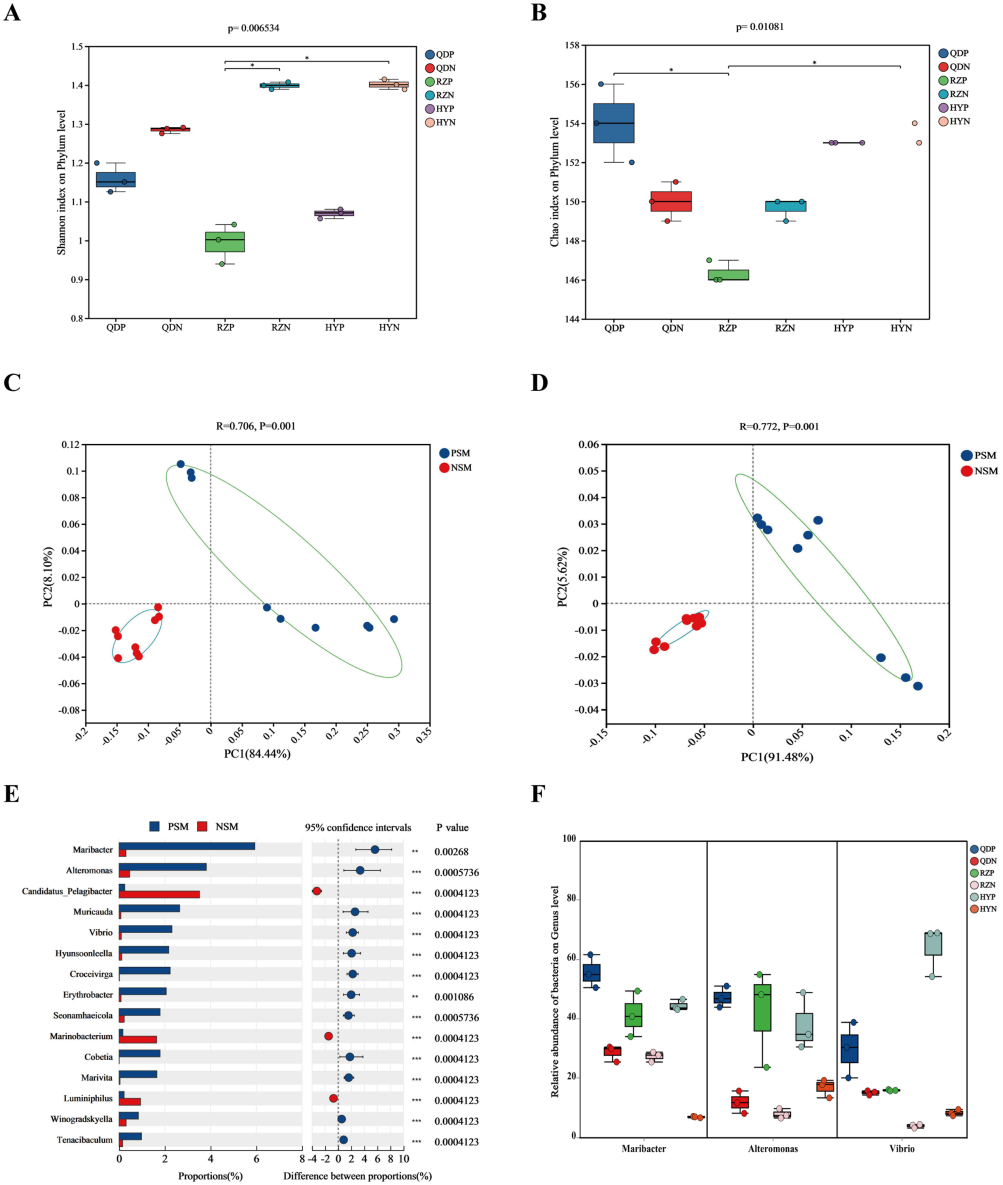
Microbial community diversity and species differences. **(A)** Shannon and **(B)** Chao1 alpha diversity indices of the bacterial communities. **(C)** PCoA of the bacterial community composition at the phylum level and **(D)** KEGG level 3 based on Bray-Curtis distance (*p* < 0.01). **(E)** Wilcoxon rank-sum test of the top 15 bacteria at the genus level and **(F)** the three bacteria with the highest difference in abundance. QDP, RZP and HYP represent phycospheric seawater microorganisms (PSM), QDN, RZN and HYN represent nonalage-covered seawater microorganisms (NSM). *p* < 0.05 *, *p* < 0.01 **, *p* < 0.001 ***.

The alpha diversity index was derived from the annotation results using the NR database, the Shannon index (Figure 3A) of the PSM group was markedly lower (*p* < 0.01), and the Chao1 richness index (Figure 3B) of the two groups significantly differed (*p* < 0.05). According to the analysis of similarities (ANOSIM), the bacterial community of the phycosphere changed significantly after the green tide reached the demise stage (*p* < 0.01). PC1 represented the PSM group and explained 84.44% of the variation, whereas PC2 explained 8.10% of the variation in the NSM group (Figure 3C). Additionally, the PSM (91.48%) and NSM (5.62%) groups presented clear functional distinctions at KEGG level 3 (Figure 3D). The Wilcoxon rank-sum test was used to analyze the top 15 bacteria in abundance (apart from unclassified bacteria) at the genus level to determine the differences in the composition of the bacterial communities in the phycosphere. The abundance of *Maribacter, Alteromonas, Muricauda, Vibrio, Hyunsoonleella, Croceivirga, Erythrobacter, Seonamhaeicola, Cobetia*, and *Marivita* in the phycosphere significantly higher during the demise stage of green tide (Figure 3E). *Maribacter*, *Alteromonas*, and *Vibrio* differed by more than 10 times in abundance between the phycospheric seawater and nonalgae-covered seawater (Figure 3F).

### 3.3 Microbial co-occurrence network analysis in the phycosphere

**Figure 4:**
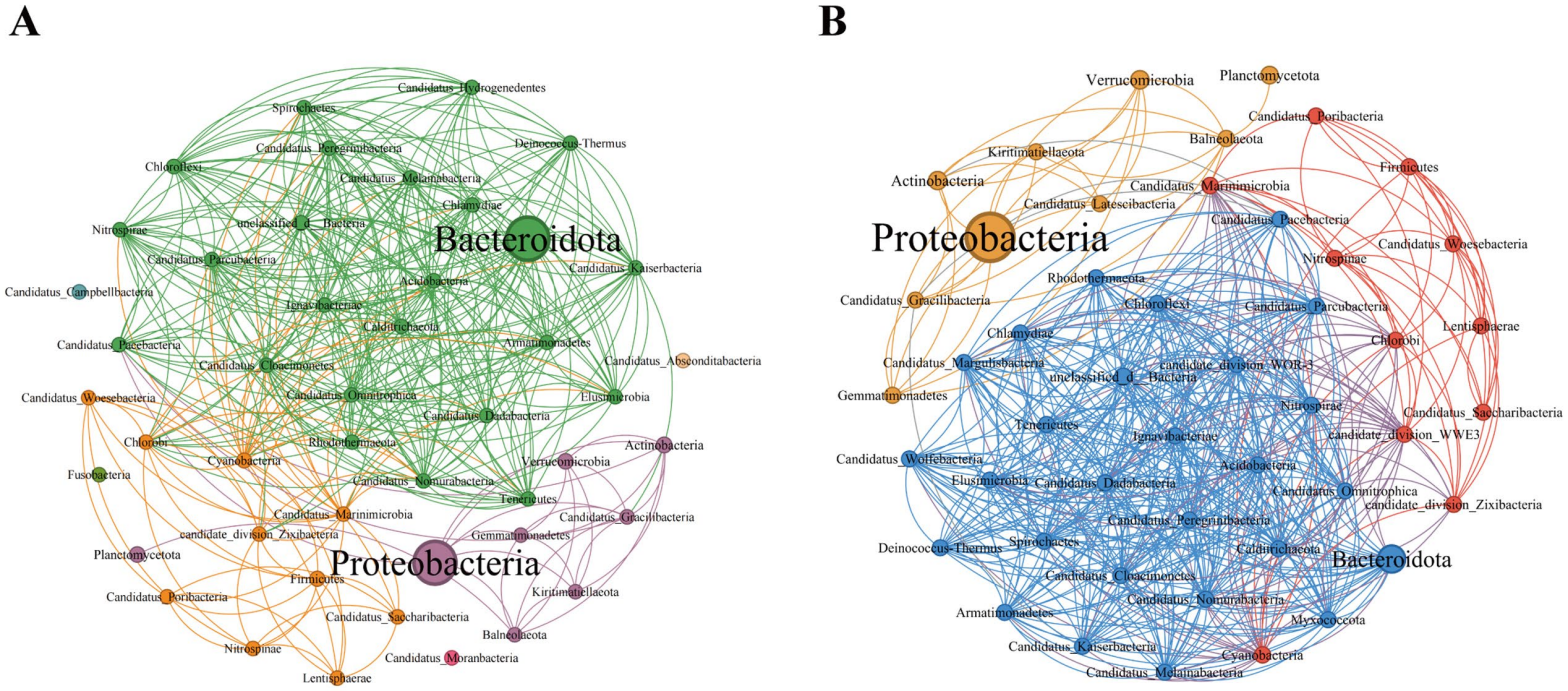
Topological characteristics of the bacterial community structure at the phylum level. The classification and coloring are based on the modularity class, and the size of the node represents the bacterial abundance. The connections represent the correlation coefficient between bacteria. **(A)** Represents the PSM network (phycospheric seawater microorganisms), and **(B)** represents the NSM network (nonalgae-covered seawater microorganisms).

On the basis of the Spearman coefficient, the top 50 dominant bacteria were selected to construct a single-factor co-occurrence network diagram (Figure 4). At the phylum level, the PSM and NSM networks contained 47 and 46 nodes and 370 and 491 edges, respectively (Supplementary Tables 2, 3). Most of the correlations in both networks were positive, and the average degree and graph density in the PSM network were lower than those in the NSM network. The PSM network was divided into seven communities, whereas the NSM network was divided into three communities. In addition, nodes with high clustering value in the PSM group were Proteobacteria, Balneolaeota, Verrucomicrobia and Deinococcus-Thermus, and in the NSM group were Rhodothermaeota, Deinococcus-Thermus, and Candidatus_Melainabacteria.

### 3.4 Microbial contributions to the element cycle in the phycosphere

**Figure 5:**
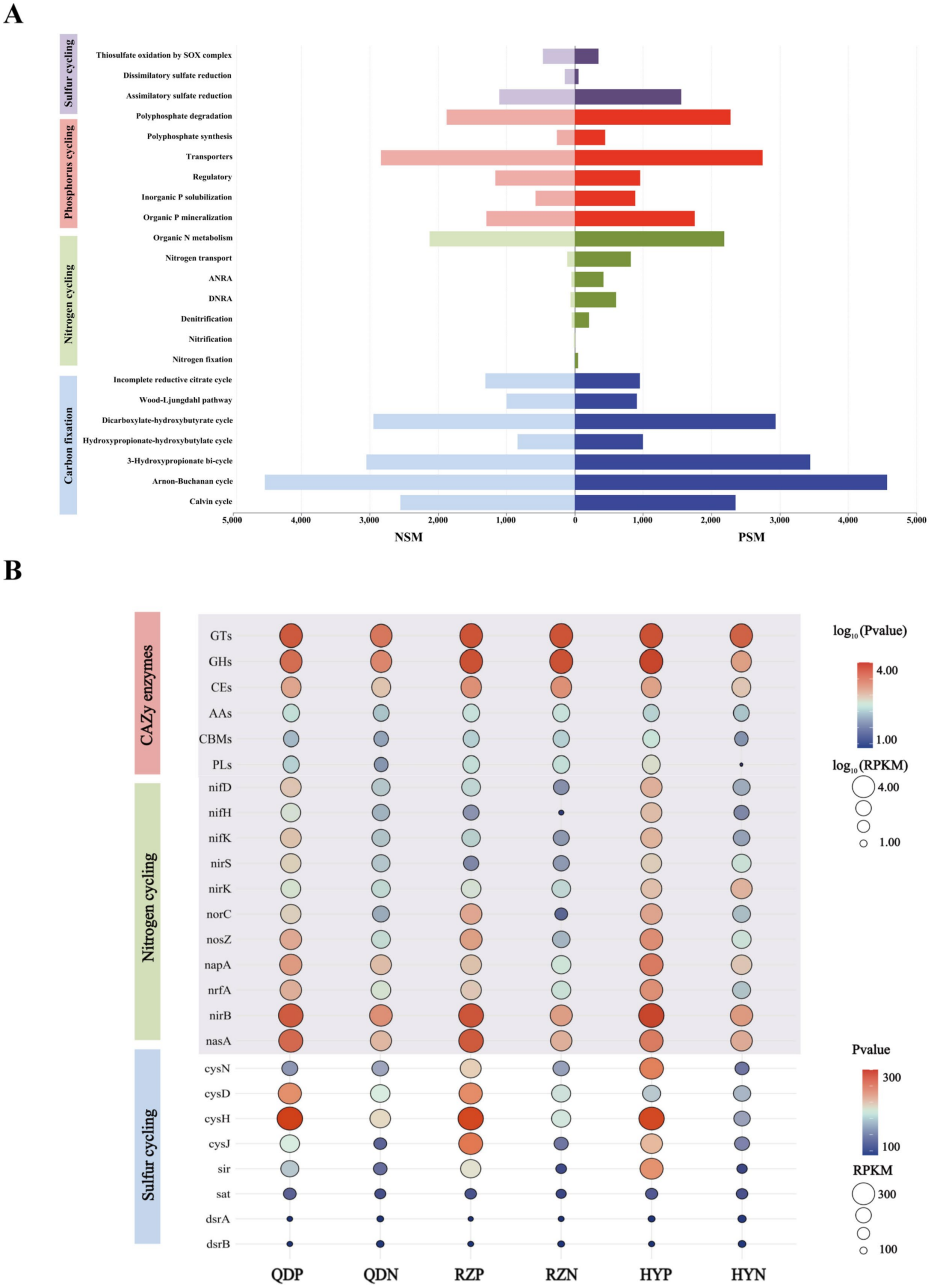
The main pathways of element cycles **(A)** and the levels of major enzymes and genes **(B)**. The abundances of six CAZy enzymes and genes associated with the nitrogen cycle were normalized using log_10_ values.

Six main carbohydrate-active enzymes (CAZy enzymes) were detected in both the PSM and NSM groups: glycosyl transferases (GTs), glycoside hydrolases (GHs), carbohydrate esterases (CEs), auxiliary active enzymes (AAs), carbohydrate-binding modules (CBMs), and polysaccharide lyases (PLs). The abundances of the six CAZy enzymes in the PSM group were significantly increased (*p* < 0.05), especially those of the GHs and PLs (Figure 5B). For the six CAZy enzymes, the contribution of Flavobacteriaceae in the PSM group was the highest, reaching more than 70%. At the phylum level, the contributions of Proteobacteria and Bacteroidota were greater than 88% in the PSM group and greater than 58% in the NSM group (Supplementary Figure 1).

The PSM and NSM groups contain a series of nitrogen transformation function, which mainly through organic N metabolism, nitrogen transport, dissimilatory nitrate reduction to ammonium (DNRA), assimilatory nitrate reduction to ammonium (ANRA), denitrification, nitrogen fixation and nitrification metabolism pathways (Figure 5A). The genes with DNRA function (*napA*, *nrfA,* and *nirB*) and genes with denitrification function (*nif D/H/K*, *nirK/S*, *norC*, and *nosZ*) in the PSM group were markedly greater, especially *norC*. The main gene with ANRA function was *nasA*, and the abundance of *nasA* in the PSM group was dramatically greater than that in the NSM group (Figure 5B).

Three pathways related to the sulfur cycle were annotated in this experiment: assimilatory sulfate reduction (ASR), dissimilatory sulfate reduction (DSR) and thiosulfate oxidation by the SOX complex (Figure 5A). The abundances of the genes *cysN/D/H/J*, *sir*, *sat*, and *dsrA*/*B* in the PSM group were significantly greater during the demise stage of the green tide (Figure 5B).

### 3.5 Potential environmental drivers for microbial community in the phycosphere

**Figure 6:**
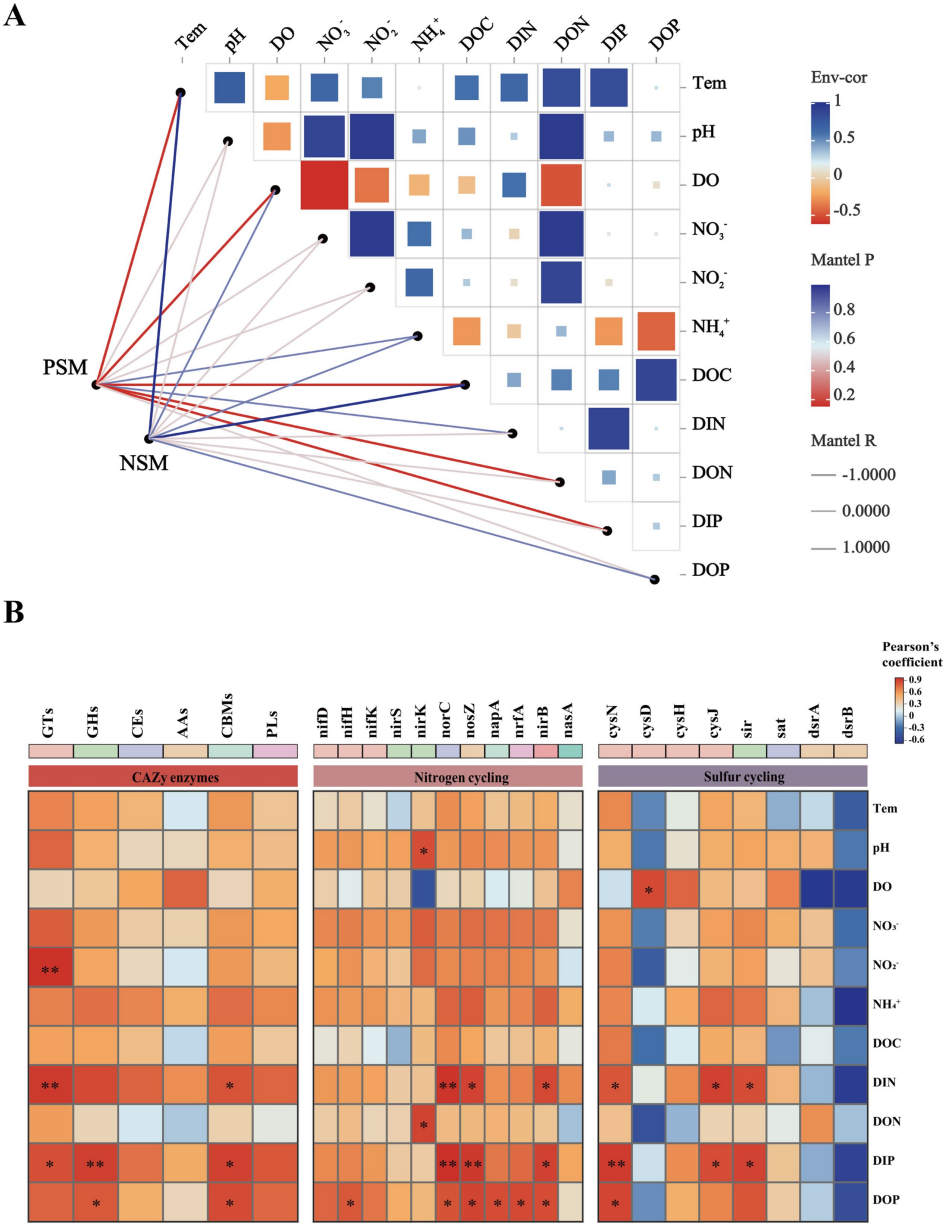
Correlation heatmaps between environmental factors and the microbial community **(A)**, functional enzymes and genes **(B)**. The size and shade of each square are proportional to the degree of correlation. PSM represents phycospheric seawater microorganisms, and NSM represents nonalgae-covered seawater microorganisms. *p* < 0.05 *, *p* < 0.01 **.

A Mantel test was performed to further explore the environmental driving factors of microbial changes in the phycosphere during the demise of *U. prolifera*. The environmental factors of seawater had a strong positive correlation with the composition of the bacterial community in the PSM group, and NO_2_^−^, NO_3_^−^, DIN, and DON had important effects on the composition of the bacterial community in the PSM group (*p* < 0.05) (Figure 6A). Pearson correlation analysis revealed that the strongest positive correlation in CAZy enzymes in the phycosphere was between DOC and GHs, followed by GTs, CEs, and PLs. There was generally a positive correlation between the genes involved in the nitrogen metabolism pathway and various nutrient elements, among which the positive correlations between *nirK* and DON, and *norB*, *nosZ*, *nirB*, and DIN were the strongest. However, there was a general negative correlation between genes involved in sulfur metabolism pathways and various other environmental factors (Figure 6B).

## 4 Discussion

This study analyzed the changes in the structure of the microbial community within the phycospheric microenvironment and biogeochemical effects induced in the late stage of *U. prolifera* green tides. Our results revealed that during the demise stage of green tides, significant changes have taken place in the structure and diversity of phycospheric microorganisms. Meanwhile, founded that this changes will further affect the element cycling process in the phycospheric microenvironment.

We found that the demise of *U. prolifera* can change the environmental factors and microbial community diversity in the phycospheric microenvironment. Generally, *U. prolifera* decomposes could release many organic compounds and consume oxygen in the late stage of green tide (Zhou et al., 2019; DeRose et al., 2021). Our study also obtained similar findings that the concentration of DO decreased, and the concentrations of DOC and nitrogenous nutrients increased significantly in the phycospheric microenvironment (Figure 1). Consistent with previous findings showing that some bacteria can use the organic matter released by algae to reproduce rapidly and gain an advantage in the competition, such as *Algoriphagus* and *Marinobacter* (Liang et al., 2021; Kim et al., 2023). Our results showed that the microbial diversity in the phycosphere decreased significantly (Figure 3). This could be the large amount of organic matter released by *U. prolifera* will promote the growth of some specific microbe rapidly, thus occupy a dominant position in the phycospheric microenvironment, ultimately lead to a decrease in microbial diversity.

There was a significant difference in the composition of the microbial community between the phycospheric microenvironment and surrounding seawater environment during the demise of *U. prolifera*. In our research, Proteobacteria and Bacteroidota at the phylum level together accounted for more than 80% of the total bacteria in the phycosphere. Flavobacteriaceae at the family level and *Alteromonas*, *Maribacter* and *Vibrio* at the genus level increased significantly during the demise stage of the green tide (Figures 2, 3). Proteobacteria and Bacteroidota are the two most common phyla in the phycosphere and exhibit a wide range of functional diversity as well as ecological adaptability (Coyne et al., 2022). Many isolated algae-lysing bacteria belong to Proteobacteria and Bacteroidota (Gao et al., 2020; Wang M. et al., 2020). In addition, relevant studies have shown that Flavobacteriaceae inhibits the growth of many HAB species, such as *Microcystis aeruginosa* and *Prococentrum marinum* (Rishiram et al., 2016). This is because the genome of Flavobacteriaceae species contains many genes encoding polysaccharide-degrading enzymes, so Flavobacteriaceae can use and decompose a variety of complex carbon sources (Gavriilidou et al., 2020). In recent years, a variety of algae-lysing bacteria in genera such as *Streptomyces*, *Maribacter*, *Pseudoalteromonas*, *Alteromonas*, *Shewanella*, and *Vibrio*, which are important organic degradation agents for complex biological macromolecules and algae fragments, have been discovered (Kim et al., 2023; Zheng et al., 2018). Attention worthy, we detected high abundance of *Maribacter*, *Alteromonas* and *Vibrio* in the phycosphere, which can promote carbon and nitrogen cycling by degrading aromatic compounds into simpler compounds (Ahmad et al., 2020). On the basis of these findings, we inferred that the significant increase in algae-lysing bacteria in the phycospheric microenvironment may participate in the degradation of algal cells, further decomposing the organic carbon and nitrogen substances released by them. Therefore, biodegradability may be an important reason for the enrichment of algae-lysing bacteria in the phycospheric microenvironment during the late stage of *U. prolifera* green tide.

Previous studies have shown that seagrass and some macroalgae surfaces can selectively regulate the adhesion and sedimentation of microorganisms in the surrounding environment to establish specific microbial communities, often referred to as the “host effect” (Roth-Schulze et al., 2016; Roth-Schulze et al., 2018). In our research, the topological network diagram revealed that *U. prolifera* in the demise stage had a similar mechanism (Figure 4). The number of nodes and edges in the phycosphere network is lower than that in the nonalgae-covered network, and the complexity of the network structure is also lower, which is consistent with the abovementioned findings that the microbial community diversity of the phycosphere decreases significantly in the late period. The lower average degree and graph density in the phycosphere network indicated that there were fewer connections in the symbiotic network of microorganisms in the phycosphere; therefore, *U. prolifera* entering the demise stage may reduce the interaction of microbial communities in the phycosphere. However, the specific regulatory mode still needs further experimental investigation.

In addition, we found that microorganisms play important roles in regulating the process of carbon, nitrogen and sulfur element transformation in the phycospheric microenvironment during the demise stage of green tide. Previous studies have found that phycospheric microorganisms can rapidly consume polysaccharides because of the presence of many polysaccharide utilization loci and specific substrate-binding proteins (Francis et al., 2021). In our research, CAZy enzymes were highly enriched in the phycosphere (Figure 5). Particularly, the abundance of GHs and PLs with decompose and utilize polysaccharides function in the phycospheric microenvironment is significantly higher than that in the surrounding seawater environment. This conclusion is consistent with the PLs enzyme play a major role in cleaving complex polysaccharides into simple oligomers or monomers (Chen et al., 2022; Mühlenbruch et al., 2018). Recent studies have demonstrated that various bacteria in the phycosphere display substrate preferences for the decomposition and utilization of polysaccharides (Lu et al., 2023; Zhao et al., 2022). Therefore, we infer that when *U. prolifera* demise, the release of a large amount of organic matter attracts microorganisms that hydrolyse or cleave this organic matter to gather and grow rapidly in seawater, which also allocates organic carbon resources. This can increase the metabolic efficiency of carbon in the phycospheric microenvironment, ultimately influencing the carbon transformation process.

Nitrogen transformation is an important part of the marine biogeochemical cycle (Zehr and Capone, 2020). In our study, genes associated with the processes of DNRA, denitrification and ANRA were found to be abundant within the phycosphere (Figure 6), highlighting the diversity of nitrogen transformation processes in this microenvironment. The *nrfA* is essential for DNRA process, facilitating the conversion of nitrate to ammonium, thus influencing nitrogen retention in ecosystems (Wang S. et al., 2020). The presence of *nirS/K* and *norC* are linked to denitrification, which convert nitrite to nitric oxide. This process effectively eliminate fixed nitrogen from the environment (Liao et al., 2024). *U. prolifera* consumes a lot of oxygen in the process of demise, which will lead to the formation of hypoxia or anaerobic environment, therefore it may be beneficial to the anaerobic metabolic process such as DNRA and denitrification (Liu et al., 2021). Additionally, the abundance of *nrfA* and *nirS* have been correlated with the rates of DNRA and denitrification, suggesting that these genes are crucial for understanding nitrogen dynamics in environment (Pandey et al., 2020). Furthermore, *nasA* involved in the ANRA process have the capacity to convert nitrite into ammonia, further contributing to nitrogen transformation (Wang et al., 2024). Previous findings showing that, a large amount of organic matter released during the decomposition of algae provide a rich source of nutrients for microorganisms and can promote the growth and reproduction of them (Broman et al., 2021). Thus for microbes involved in DNRA, denitrification and ANRA processes, the increase of nutrients may promote their activity and further increase the abundance of related genes. Consequently, the form and distribution of nitrogen will be altered, the process of nitrogen transformation will be accelerated, and the nitrogen cycle within the phycospheric microenvironment will be significantly impacted.

**Figure 7:**
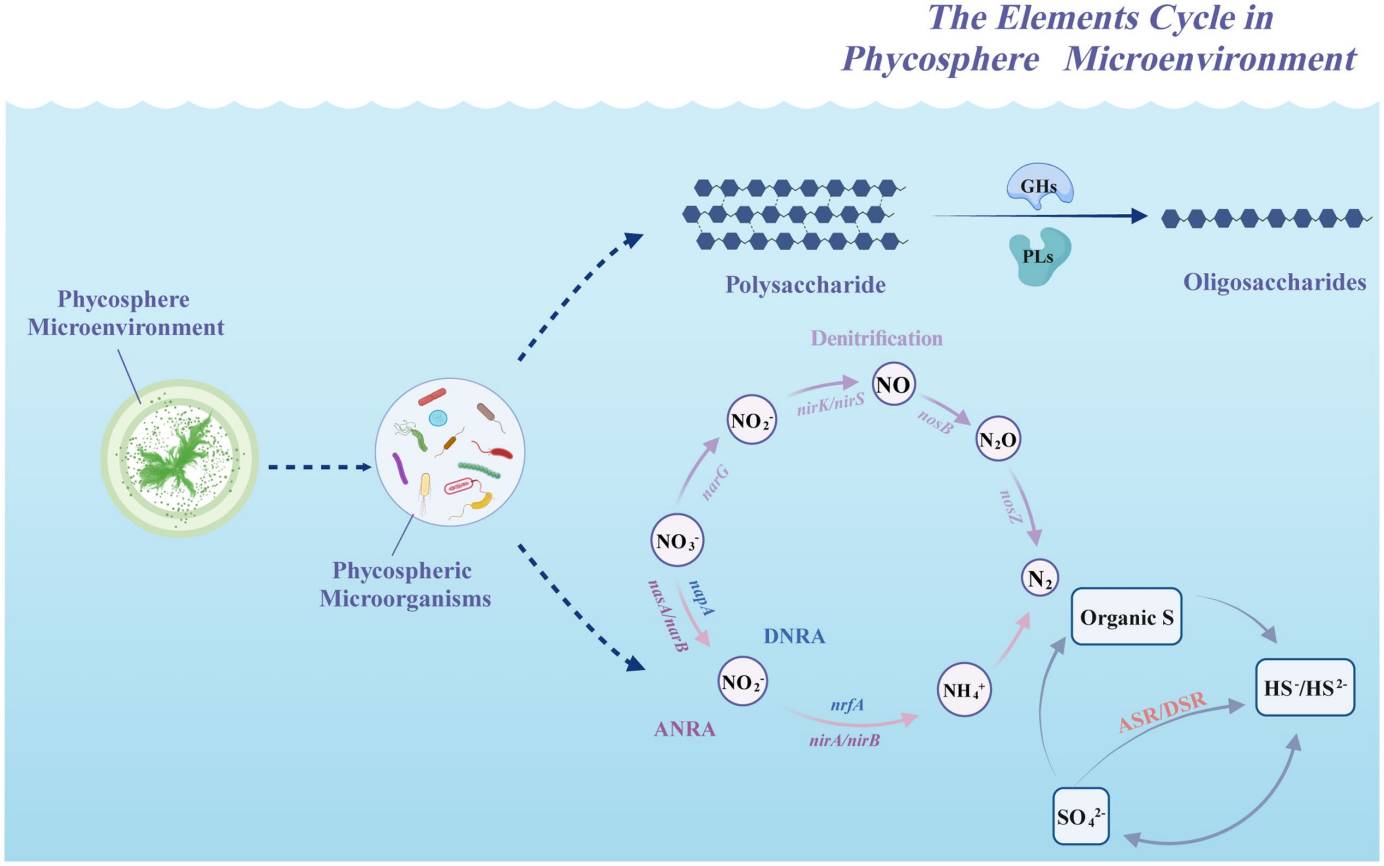
Schematic processes of the elements cycle driven by microorganisms in the phycospheric microenvironment.

The abundances of the genes *cysN/D/H/J*, *sir*, *sat* and *dsrA/B* in the phycosphere were relatively high (Figure 5), indicating that the ASR and DSR pathways were significantly activated in the phycosphere. These two pathways are the core pathways of the marine sulfur cycle (Wang S. et al., 2023), and genes involved in these pathways can assimilate sulfide and convert it into cysteine, which is closely related to carbohydrate metabolism, carbon and nitrogen balance, protein synthesis and secondary metabolism, etc. (Sakurai et al., 2010). Thus, the sulfur reduction process is active in the phycospheric microenvironment, and there is a close relationship between the sulfur cycle and the carbon and nitrogen cycles (Figure 7).

## 5 Conclusion

Our research indicates that after *U. prolifera* green tides enter the demise stage, they can affect the phycospheric microenvironment, resulting in a distinct microbial community composition and structure. A large proportion of algae-lysing bacteria in the phycosphere also have unique functions and are crucial for niche adaptation and interactions. In addition, phycospheric microorganisms can increase the efficiency of carbon and nitrogen metabolism and thus significantly influence marine biogeochemical processes. Therefore, *U. prolifera* green tides during the demise stage could affect potential biogeochemical processes in the Yellow Sea by driving changes in phycospheric microbes. However, the role of microorganisms in this process within such a large-scale green tide, laden with organic biogenic elements, warrants further investigation through additional experiments.

## Data Availability

The authors acknowledge that the data presented in this study must be deposited and made publicly available in an acceptable repository, prior to publication. Frontiers cannot accept a manuscript that does not adhere to our open data policies.

## Footnotes

^1^ https://github.com/OpenGene/fastp
