# Supplementary material for: Changes in the structure of the microbial community within the phycospheric microenvironment and potential biogeochemical effects induced in the demise stage of green tides caused by Ulva prolifera
Source: Front Microbiol. 2024 Nov 5;15:1507660. doi: 10.3389/fmicb.2024.1507660 (PMC11575915; doi:10.3389/fmicb.2024.1507660)
Supplement: Supplementary file 1 [file Supplementary_file_1.docx]

Supplementary Material

# Supplementary Figures and Tables

## Supplementary Figures


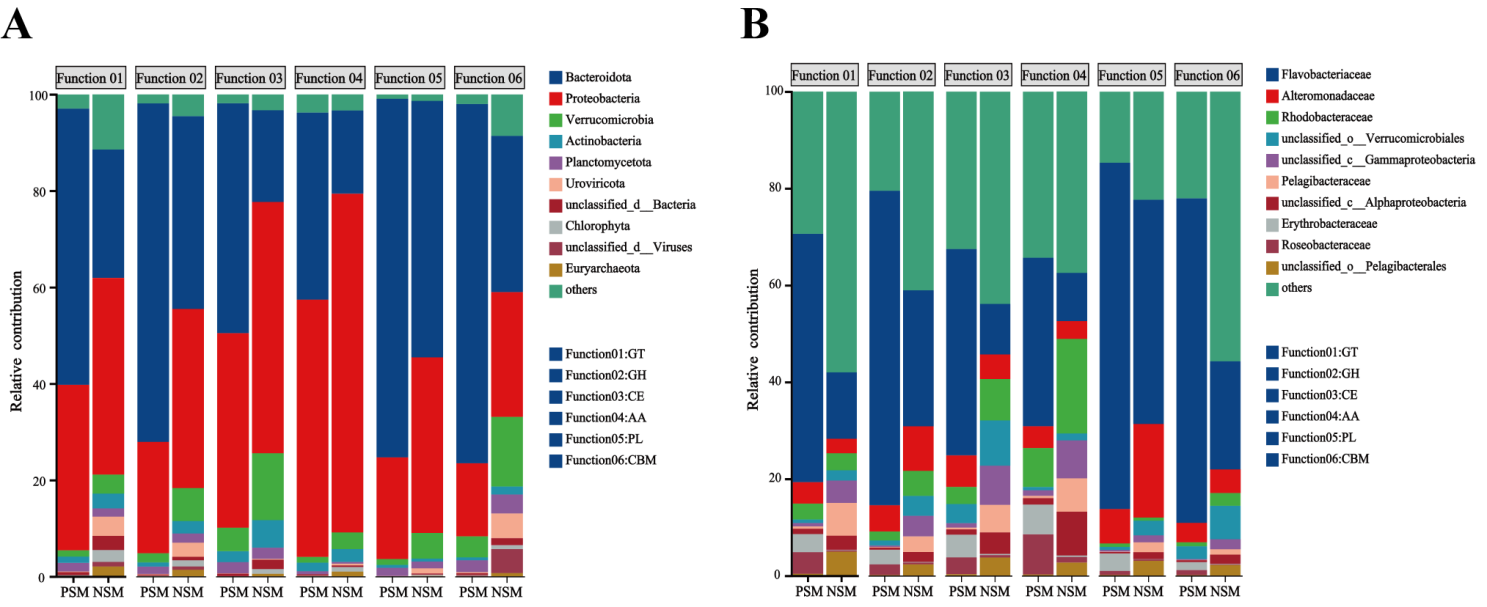


**Supplementary Figure 1.** Contribution bar plot of species and CAZy enzymes at the phylum **(A)** and family **(B)** levels. PSM represents phycospheric seawater microorganisms, and NSM represents nonalgae-covered seawater microorganisms.

## Supplementary Tables

**Supplementary Table 1.** Statistical table of data after each sample was hosted.

| Samples | Optimized reads | Optimized bases (bp) | Percent in raw reads (%) | Percent in raw bases (%) |
| --- | --- | --- | --- | --- |
| QD1 | 95641902 | 14358779867 | 96.03638104 | 95.48346528 |
| QD2 | 95089688 | 14280184664 | 96.05446481 | 95.53027114 |
| QD3 | 83652998 | 12566376608 | 96.38492184 | 95.88721686 |
| RZ1 | 88721776 | 13326316194 | 96.40132956 | 95.89279190 |
| RZ2 | 100740626 | 15114174317 | 95.68424017 | 95.06994589 |
| RZ3 | 101494946 | 15214740232 | 95.62687546 | 94.93429804 |
| HY1 | 83927902 | 12588367931 | 96.19010942 | 95.54688492 |
| HY2 | 86728110 | 13003106570 | 96.15040339 | 95.46878666 |
| HY3 | 80910832 | 12144740402 | 96.49361710 | 95.91868323 |
| CD1 | 95400966 | 14275601402 | 95.67731461 | 94.81426246 |
| CD2 | 92685396 | 13857297381 | 95.42023537 | 94.47801574 |
| CD3 | 114286214 | 17121594480 | 95.76622346 | 95.01360029 |
| CZ1 | 90094202 | 13477782685 | 94.69446478 | 93.81434551 |
| CZ2 | 84278362 | 12634403086 | 95.42030472 | 94.73326248 |
| CZ3 | 96246294 | 14426907842 | 95.42175651 | 94.72392847 |
| CT1 | 109230916 | 16247203224 | 94.54661793 | 93.13267732 |
| CT2 | 96700336 | 14474166339 | 94.96731358 | 94.13752627 |
| CT3 | 96043026 | 14374483217 | 95.02358824 | 94.18481392 |

**Supplementary Table 2.** PSM network centre coefficient with single factor correlation.

| Node_ID | Node_Name | Degree_Centrality | Closeness_Centrality | Betweenness_Centrality |
| --- | --- | --- | --- | --- |
| 1 | Bacteroidota | 0.102040816 | 0.445454545 | 0.063010736 |
| 2 | Proteobacteria | 0.040816327 | 0.365671642 | 0 |
| 3 | Cyanobacteria | 0.469387755 | 0.597560976 | 0.04100014 |
| 4 | Acidobacteria | 0.469387755 | 0.597560976 | 0.04100014 |
| 5 | Rhodothermaeota | 0.163265306 | 0.457943925 | 0.048678057 |
| 6 | Deferribacteres | 0.020408163 | 0.310126582 | 0 |
| 7 | Actinobacteria | 0.428571429 | 0.597560976 | 0.014478752 |
| 8 | Verrucomicrobia | 0.367346939 | 0.532608696 | 0.000462803 |
| 9 | Firmicutes | 0.367346939 | 0.569767442 | 0.012366291 |
| 10 | Chloroflexi | 0.448979592 | 0.597560976 | 0.005733108 |
| 11 | Candidatus_Pacebacteria | 0.551020408 | 0.644736842 | 0.041634052 |
| 12 | Candidatus_Marinimicrobia | 0.469387755 | 0.6125 | 0.013858505 |
| 13 | Candidatus_Kaiserbacteria | 0.469387755 | 0.604938272 | 0.00988396 |
| 14 | Candidatus_Saccharibacteria | 0.408163265 | 0.544444444 | 0.005342482 |
| 15 | Deinococcus-Thermus | 0.367346939 | 0.532608696 | 0.000462803 |
| 16 | Ignavibacteriae | 0.469387755 | 0.604938272 | 0.007064852 |
| 17 | Spirochaetes | 0.551020408 | 0.662162162 | 0.047311354 |
| 18 | Gemmatimonadetes | 0.510204082 | 0.644736842 | 0.020392869 |
| 19 | Chlorobi | 0.510204082 | 0.644736842 | 0.020392869 |
| 20 | Candidatus_Woesebacteria | 0.428571429 | 0.590361446 | 0.004518786 |
| 21 | Kiritimatiellaeota | 0.510204082 | 0.644736842 | 0.020392869 |
| 22 | Candidatus_Nomurabacteria | 0.448979592 | 0.6125 | 0.012244116 |
| 23 | Tenericutes | 0.183673469 | 0.521276596 | 0.006071132 |
| 24 | Lentisphaerae | 0.469387755 | 0.604938272 | 0.007064852 |
| 25 | Candidatus_Omnitrophica | 0.571428571 | 0.653333333 | 0.058919436 |
| 26 | candidate_division_Zixibacteria | 0.469387755 | 0.604938272 | 0.007064852 |
| 27 | Candidatus_Falkowbacteria | 0.387755102 | 0.521276596 | 0.030336368 |
| 28 | Planctomycetota | 0.408163265 | 0.556818182 | 0.000449119 |
| 29 | unclassified_d__Bacteria | 0.632653061 | 0.7 | 0.056821967 |
| 30 | Candidatus_Gracilibacteria | 0.428571429 | 0.563218391 | 0.005934131 |
| 31 | Candidatus_Peregrinibacteria | 0.387755102 | 0.538461538 | 0.001262025 |
| 32 | Chlamydiae | 0.448979592 | 0.6125 | 0.005580557 |
| 33 | Nitrospirae | 0.448979592 | 0.6125 | 0.005580557 |
| 34 | Candidatus_Dadabacteria | 0.469387755 | 0.597560976 | 0.007013673 |
| 35 | Candidatus_Campbellbacteria | 0.428571429 | 0.583333333 | 0.0045026 |
| 36 | Nitrospinae | 0.489795918 | 0.628205128 | 0.014220552 |
| 37 | Armatimonadetes | 0.448979592 | 0.6125 | 0.005580557 |
| 38 | Candidatus_Parcubacteria | 0.510204082 | 0.636363636 | 0.014297192 |
| 39 | Candidatus_Absconditabacteria | 0.448979592 | 0.590361446 | 0.013242047 |
| 40 | Elusimicrobia | 0.510204082 | 0.636363636 | 0.014297192 |
| 41 | Candidatus_Hydrogenedentes | 0.632653061 | 0.7 | 0.053563778 |
| 42 | Candidatus_Cloacimonetes | 0.591836735 | 0.680555556 | 0.054971834 |
| 43 | Candidatus_Moranbacteria | 0.428571429 | 0.583333333 | 0.00186473 |
| 44 | Fusobacteria | 0.408163265 | 0.576470588 | 0.012806313 |
| 45 | Fibrobacteres | 0.408163265 | 0.550561798 | 0.001611632 |
| 46 | Candidatus_Poribacteria | 0.12244898 | 0.411764706 | 0.000261233 |
| 47 | Calditrichaeota | 0.224489796 | 0.449541284 | 0.001421636 |
| 48 | Candidatus_Melainabacteria | 0.285714286 | 0.480392157 | 0.016008024 |
| 49 | Candidatus_Latescibacteria | 0.346938776 | 0.563218391 | 0.006607502 |
| 50 | Balneolaeota | 0.040816327 | 0.34751773 | 0 |

**Supplementary Table 3.** NSM network centre coefficient with single factor correlation.

| Node_ID | Node_Name | Degree_Centrality | Closeness_Centrality | Betweenness_Centrality |
| --- | --- | --- | --- | --- |
| 1 | Proteobacteria | 0.163265306 | 0.422413793 | 0.025306454 |
| 2 | Verrucomicrobia | 0.163265306 | 0.355072464 | 0.000121477 |
| 3 | Actinobacteria | 0.183673469 | 0.426086957 | 0.025897915 |
| 4 | Balneolaeota | 0.183673469 | 0.357664234 | 0.040937804 |
| 5 | Gemmatimonadetes | 0.163265306 | 0.355072464 | 0.000121477 |
| 6 | Kiritimatiellaeota | 0.163265306 | 0.355072464 | 0.000121477 |
| 7 | Candidatus_Gracilibacteria | 0.204081633 | 0.49 | 0.11794253 |
| 8 | Candidatus_Pacebacteria | 0.448979592 | 0.590361446 | 0.145029286 |
| 9 | Candidatus_Handelsmanbacteria | 0.163265306 | 0.355072464 | 0.000121477 |
| 10 | Bacteroidota | 0.612244898 | 0.628205128 | 0.003388995 |
| 11 | unclassified_d__Bacteria | 0.653061224 | 0.671232877 | 0.009605277 |
| 12 | Chloroflexi | 0.632653061 | 0.653333333 | 0.006099638 |
| 13 | Cyanobacteria | 0.612244898 | 0.628205128 | 0.057144351 |
| 14 | Acidobacteria | 0.673469388 | 0.680555556 | 0.013832902 |
| 15 | Spirochaetes | 0.632653061 | 0.662162162 | 0.007590517 |
| 16 | Tenericutes | 0.591836735 | 0.620253165 | 0.002032506 |
| 17 | Ignavibacteriae | 0.653061224 | 0.671232877 | 0.009605277 |
| 18 | Rhodothermaeota | 0.489795918 | 0.505154639 | 0 |
| 19 | Candidatus_Dadabacteria | 0.653061224 | 0.671232877 | 0.009605277 |
| 20 | Chlamydiae | 0.632653061 | 0.653333333 | 0.006099638 |
| 21 | Candidatus_Peregrinibacteria | 0.632653061 | 0.653333333 | 0.007264579 |
| 22 | candidate_division_WOR-3 | 0.673469388 | 0.680555556 | 0.013832902 |
| 23 | Candidatus_Margulisbacteria | 0.612244898 | 0.644736842 | 0.005642881 |
| 24 | Deinococcus-Thermus | 0.530612245 | 0.563218391 | 0.000812008 |
| 25 | Candidatus_Kaiserbacteria | 0.591836735 | 0.690140845 | 0.110980094 |
| 26 | Nitrospirae | 0.653061224 | 0.662162162 | 0.010327263 |
| 27 | Candidatus_Omnitrophica | 0.653061224 | 0.662162162 | 0.010700648 |
| 28 | Myxococcota | 0.591836735 | 0.620253165 | 0.002032506 |
| 29 | Chlorobi | 0.428571429 | 0.521276596 | 0.021610578 |
| 30 | Candidatus_Wolfebacteria | 0.551020408 | 0.576470588 | 0.001244997 |
| 31 | candidate_division_WWE3 | 0.612244898 | 0.628205128 | 0.066409043 |
| 32 | Elusimicrobia | 0.591836735 | 0.6125 | 0.00323289 |
| 33 | Candidatus_Nomurabacteria | 0.653061224 | 0.662162162 | 0.010700648 |
| 34 | Candidatus_Melainabacteria | 0.571428571 | 0.604938272 | 0.000823862 |
| 35 | Armatimonadetes | 0.571428571 | 0.590361446 | 0.0014127 |
| 36 | Candidatus_Cloacimonetes | 0.632653061 | 0.662162162 | 0.007590517 |
| 37 | Candidatus_Parcubacteria | 0.632653061 | 0.662162162 | 0.007590517 |
| 38 | Calditrichaeota | 0.653061224 | 0.662162162 | 0.010327263 |
| 39 | Candidatus_Hydrogenedentes | 0.428571429 | 0.583333333 | 0.066220047 |
| 40 | Candidatus_Jacksonbacteria | 0.591836735 | 0.636363636 | 0.007217571 |
| 41 | Candidatus_Latescibacteria | 0.163265306 | 0.426086957 | 0.039883978 |
| 42 | Planctomycetota | 0.020408163 | 0.264864865 | 0 |
| 43 | Candidatus_Marinimicrobia | 0.448979592 | 0.52688172 | 0.053925591 |
| 44 | Nitrospinae | 0.183673469 | 0.426086957 | 0.002342526 |
| 45 | Candidatus_Poribacteria | 0.142857143 | 0.418803419 | 0.001244501 |
| 46 | Candidatus_Woesebacteria | 0.183673469 | 0.426086957 | 0.002342526 |
| 47 | Candidatus_Saccharibacteria | 0.12244898 | 0.365671642 | 0.000281293 |
| 48 | candidate_division_Zixibacteria | 0.306122449 | 0.49 | 0.014279699 |
| 49 | Firmicutes | 0.163265306 | 0.429824561 | 0.002493849 |
| 50 | Lentisphaerae | 0.142857143 | 0.422413793 | 0.001764305 |
